# Une réponse complète d'un dermatofibrosarcome de Darrier et Ferrand localement avancé: à propos d'un cas et revue de la littérature

**DOI:** 10.11604/pamj.2019.32.97.10293

**Published:** 2019-02-28

**Authors:** Hajar Ouahbi, Lamiae Amadour, Fatimazahra Elmernissi, Layla Tahiri, Taoufik Harmouch, Zineb Benbrahim, Fatimazahra Elmrabet, Samia Arifi, Ayat Allah Oufkigh, Nawfal Mellas

**Affiliations:** 1 Service d'Oncologie Médicale CHU Hassan II, Université Sidi Mohammed Ben Abdellah, Fès, Maroc; 2 Service de Dermatologie CHU Hassan II, Université Sidi Mohammed Ben Abdellah, Fès, Maroc; 3 Servie d'Anatomopathologie CHU Hassan II, Université Sidi Mohammed Ben Abdellah, Fès, Maroc; 4 Service de Chirurgie Maxillo-faciale et Réparatrice, CHU Hassan II, Université Sidi Mohammed Ben Abdellah, Fès, Maroc

**Keywords:** Dermato-fibrosarcome protubérant, imatinib, réponse, Erotuberant dermatofibrosarcoma, imatinib, response

## Abstract

Le dermatofibrosarcome de Darier et Ferrand (DFS) est une tumeur cutanée rare, caractérisé par son agressivité locale et son important potentiel aux récidives, dont le pronostic dépend essentiellement de la qualité de sa prise en charge. Une réponse complète avec des marges saines d'un DFS qui a été jugé initialement inopérable, après 1 an d'imatinib en situation néoadjuvante est rarement décrite dans littérature. Nous rapportons dans notre article, le cas d'une jeune patiente prise en charge au service d'oncologie médicale de Fès pour une DFS localement avancé présentant une réponse complète, afin d'évaluer la place de l'imatinib et des thérapies ciblées dans le traitement de DFS.

## Abstract (en)

Darier and Ferrand dermatofibrosarcoma (DFS) is a rare skin tumor characterized by its local aggressiveness and a high risk of recurrences. Prognosis depends essentially on the quality of treatment. Complete response in Darier and Ferrand dermatofibrosarcoma with healthy margins, initially judged inoperable after 1 year of neoadjuvant imatinib-based treatment, has been rarely described in literature. We here report the case of a young female patient treated in the Department of Medical Oncology in FES for locally advanced DFS with complete response, in order to assess the role of imatinib and of targeted therapies in the treatment of DFS.

## Introduction

Le dermato-fibrosarcome (DFS) de Darier et Ferrand réalise une tumeur fibreuse de la peau d'un type particulier. Rare, apparaissant à tout âge mais surtout à l'âge adulte sans distinction de sexe ni de race, il se caractérise par des métastases exceptionnelles, une propension élevée à la récidive et une possibilité de transformation en un sarcome vrai acquérant de ce fait la capacité de disséminer [1] .

## Patient et observation

**Figure 1:**
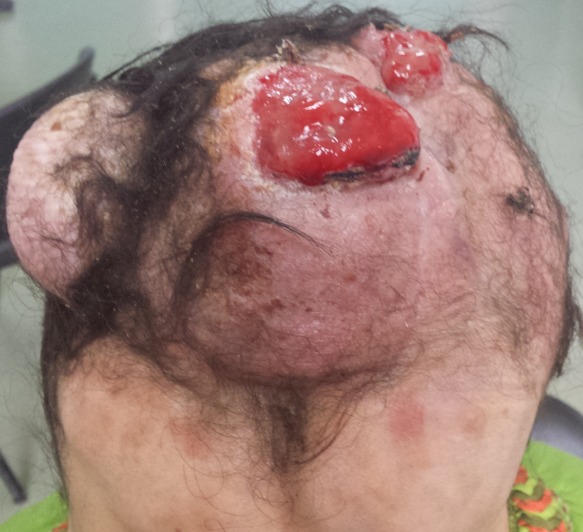
Vue supérieure d’énorme masses du cuir chevelu saignant au contact

**Figure 2:**
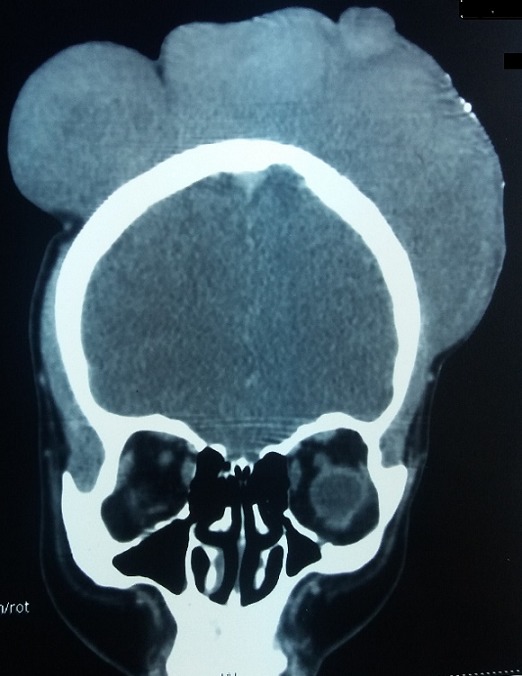
Un scanner du crâne montrant en vue antérieure - fenêtre parenchymateuse- le processus tissulaire frontopariétale

**Figure 3:**
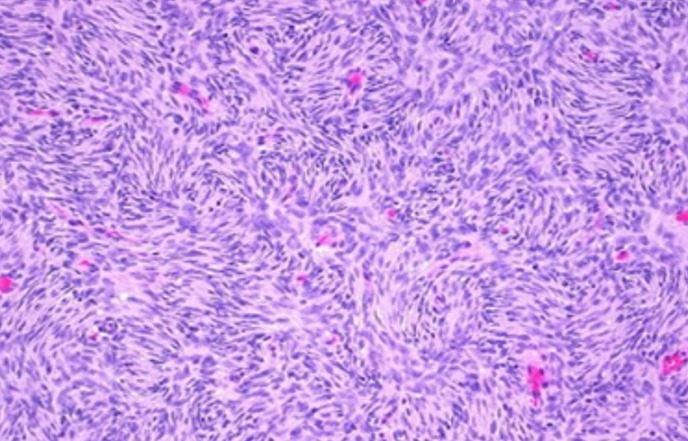
Prolifération tumorale fusocellulaire, faite de cellules fusocellulaires non atypiques (HES x 20)

**Figure 4:**
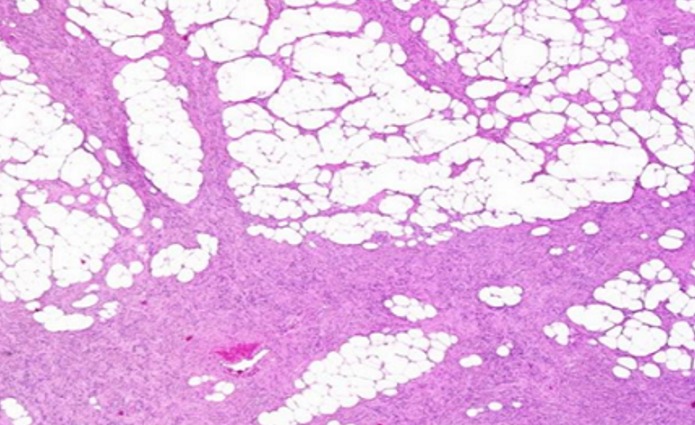
Dermatofibrosacrome infiltratrant l’hypoderme (HES x 20)

**Figure 5:**
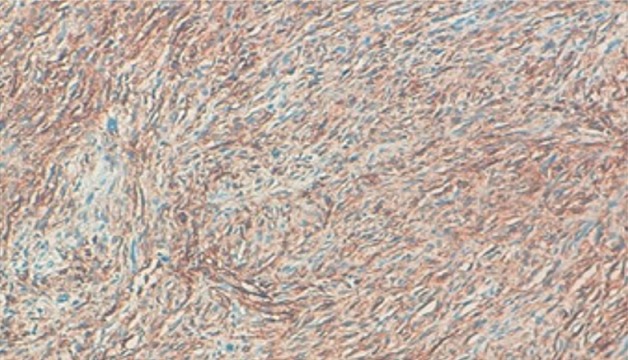
Dermatofibrosarcome de Darrier Ferrand: les cellules tumorale expriment fortement l’anticorps anti-CD34

**Figure 6:**
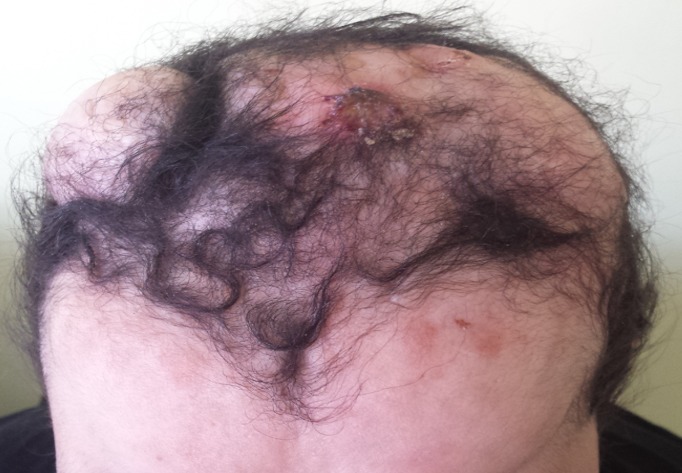
Vue supérieur frontopariétale montrant la régression quasi complete des masses tumorales sous imatinib

**Figure 7:**
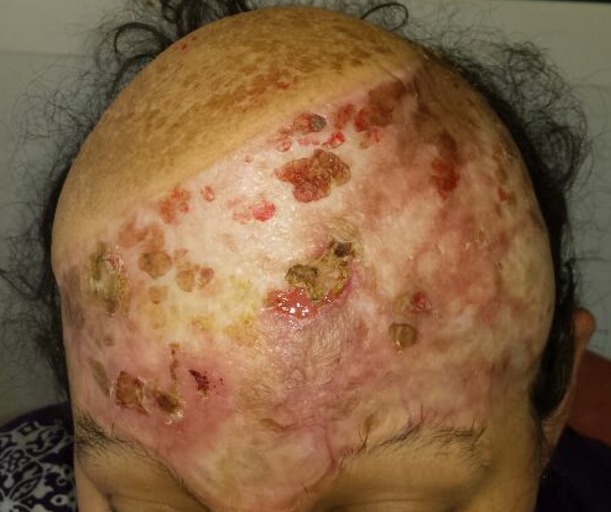
Scanner du crâne montrant en vue antérieure,le processus tissulaire frontopariétale

Mme H.G, 22 ans, deuxième geste, deuxième parité, femme au foyer, admise au service d'oncologie médicale du Centre hospitalier universitaire Hassan II de Fes (Maroc) en Janvier 2014 pour prise en charge d'une tumeur de cuir chevelu. Le debut de la symptomatologie remontait à 6 ans, par l'apparition d'un nodule cutané du cuir chevelu pariétal droit d'à peine 3 cm. Une exérèse a été réalisée dans un centre de santé sans demande d'un examen anatomopathologique.4 ans après l'exérèse , au cours de sa première grossesse ,serait survenu la première récidive au même endroit que la tumeur initial, une exérèse fut encore réalisée.une deuxième récidive est survenu deux ans plus tard ,au cours de sa deuxième grossesse.avec appariiton d'autre masse de cuir chevelu saignant au contact ,motivant sa consultation au service de dermatologie du centre hospitalier Hassan II de Fes. L'examen initial à son admission a objectivé une patiente stable sur le plan hémodynamique et respiratoire, avec à l'examen cutanée, présence de plusieurs masses de cuir chevelu, occupant les régions pariétales et frontales, protubérante, multi nodulaire, ulcéro nécrotique avec des points de saignement spontanés, la plus grande mesure 7cm de grande axe Figure 1. Un scanner crânien était demandé a objectivé un grand processus tumoral se développant au niveau des parties molles en sous cutanées de la région fronto-pariétale cranienne sans lyse osseuse en regard Figure 2. Le bilan biologique ainsi que le bilan d'extension (Radio thoracique et échographie abdominale) étaient sans particularité à part un taux d'hémoglobine à 10g/dl. L'examen anatomopathologique de la biopsie à cheval sur la peau saine et sur la tumeur avait conclu à un dermatofibro-sarcome de Darier et Ferrand (Figure 3, Figure 4, Figure 5). En réunion de concertation pluridisciplinaire, la décision d'un traitement médical en prmier chirurgie était prise.la patiente met sous imatinib à la dose de 400 mg/j, Après 1 an, elle a présenté une réduction de volume tumoral d'environ 75 % (Figure 6), permettant finalement une prise en charge chirurgicale avec des marges saines associée à une chirurgie réparatrice (Figure 7). Après un recul de 2 ans, la patiente est toujours en rémission complète.

## Discussion

Le dermatofibrosarcome de Darrier et Ferrand ou dermatofibrosarcome protuberans (DFSP) est une tumeur mésenchymateuse rare, développée aux dépens du derme et représentant moins de 2 % de l'ensemble des sarcomes des tissus mous, avec une incidence estimée à environ quatre cas pour un million [1]. Les envahissements ganglionnaires sont absents, et les métastases viscérales sont exceptionnelles [2]. C'est un cancer de bon pronostic, car en dépit des récidives locales, le pronostic Vital n'est qu'exceptionnellement engagé [2]. Pour les formes localisées, le traitement de référence est chirurgical à type d'exérèse large avec contrôle histologique strict des berges (technique de Mohs ou technique verticale modifiée) permettant de rapporter, dans les plus récentes séries, des taux de récidive après chirurgie inférieurs à 10 % [3-5]. Cependant, certains cas peuvent ne pas être accessibles à la chirurgie du fait de leurs localisations, taille, conséquences fonctionnelle et/ou esthétique prévisibles postchirurgie, extension métastatique à distance, etc. C'est pour ces situations « inopérables » qu'un traitement médical doit être envisagé, traitement reposant actuellement sur les caractéristiques moléculaires et cytogénétiques du DFSP. En effet, le DFSP est caractérisé, dans plus de 90 % des cas, par la présence d'un réarrangement réciproque entre les chromosomes 17 et 22 sous la forme soit d'une translocation t(17;22) (q22;q13). Ce réarrangement aboutit à la formation d'un gène de fusion COL1A1 (localisé sur 17q22)-PDGFB (localisé sur 22q13). La formation de ce transcrit de fusion aboutit à une surexpression de PDFGB aboutissant elle-même à une activation autocrine et paracrine permanente du récepteur du PDGFB appelé PDGFR' et donc à un signal de prolifération cellulaire. La principale cible actuelle des thérapies utilisées dans le traitement des DFSP inopérables est le PDGFR' activé par son ligand exprimé de façon aberrante [6].

L'imatinib mésylate (STI571) est une des thérapies ciblées majeures du DFSP, La place de l'imatinib dans la prise en charge de ces pathologies dérive directement de son mécanisme d'action : thérapie inhibitrice de tyrosine-kinase ciblant BCR/ABL (indication dans la LMC), KIT (indication dans les GIST), FMS (récepteur pour le colony stimulating factor 1), et PDGFR alpha et bêta (DFSP) [7]. Les travaux de Greco et al. ont porté sur les effets in vitro et in vivo de l'imatinib sur des lignées cellulaires transformées par le transcrit de fusion COL1A1-PDGFB [8]. L'utilisation de l'imatinib aboutit au blocage de la prolifération cellulaire et à l'apoptose des lignées transformées. C'est sur la base de ce rationnel scientifique qu'un certain nombre de cas cliniques, voire de petites séries de patients, ont été rapportés dans la littérature portant sur l'utilisation de l'imatinib dans les DFSP inopérables [7]. L'imatinib fut étudié dans le cadre de trois essais thérapeutiques de phase II ouverts non randomisés, permettant d'avoir une idée plus juste de l'intérêt de la molécule [9]. Ces études nous ont amenés à traiter par imatinib en première intention notre patiente avec un DFSP inopérable, dont la réponse a été spectaculaire (Figure 4, Figure 5). Les taux de réponse et les délais jusqu'à progression tumorale ne différaient pas significativement entre ces essais menés à des dosages différents (400 mg par jour ou 400 mg × 2 par jour), suggérant ainsi que 400 mg par jour est aussi efficace que 800 mg par jour [9]. La place et la durée optimale de traitement par imatinib en néoadjuvant ne sont donc pas encore définitivement établies. Des études futures seront nécessaires afin de préciser sa place en préopé- ratoire mais aussi en adjuvant après exérèse chirurgicale incomplète. Des stratégies alternatives avec de nouvelles molécules sont en cours, telles que le pazopanib, qui est actuellement évalué dans le cadre d'une autre étude multicentrique du groupe de cancérologie cutané français. Cette étude évalue l'intérêt du pazopanib dont l'efficacité sur PDGFR' est au moins égale in vitro à celle de l'imatinib, et donc susceptible de lever les résistances secondaires à cette dernière et par ailleurs doué d'activité antiangiogénique [7, 10].

## Conclusion

Une bonne connaissance du dermatofi- brosarcome de Darier et Ferrand par les praticiens et une demande systématique de l'examen histologique devant toute tumeur cutanée banale permettraient d'éviter sa confusion avec une lésion banale. Il en résultera un diagnostic précoce et un traitement adéquat, dont l'imatinib mésylate est la seule molécule qui a montré son efficacité pour les DFS inopérables. Incitant ainsi à d'évaluer des stratégies alternatives avec de nouvelles molécules.

## Conflits d'intérêts

Les auteurs ne déclarent aucun conflit d'intérêts.
